# Image Sand–Dust Removal Using Reinforced Multiscale Image Pair Training

**DOI:** 10.3390/s25195981

**Published:** 2025-09-26

**Authors:** Dong-Min Son, Jun-Ru Huang, Sung-Hak Lee

**Affiliations:** School of Electronic and Electrical Engineering, Kyungpook National University, 80 Daehak-ro, Buk-gu, Daegu 41566, Republic of Korea; forhollow@knu.ac.kr (D.-M.S.); huangjunru@knu.ac.kr (J.-R.H.)

**Keywords:** CycleGAN, Retinex, multi-scale image, color preservation

## Abstract

This study proposes an image-enhancement method to address the challenges of low visibility and color distortion in images captured during yellow sandstorms for an image sensor based outdoor surveillance system. The technique combines traditional image processing with deep learning to improve image quality while preserving color consistency during transformation. Conventional methods can partially improve color representation and reduce blurriness in sand–dust environments. However, they are limited in their ability to restore fine details and sharp object boundaries effectively. In contrast, the proposed method incorporates Retinex-based processing into the training phase, enabling enhanced clarity and sharpness in the restored images. The proposed framework comprises three main steps. First, a cycle-consistent generative adversarial network (CycleGAN) is trained with unpaired images to generate synthetically paired data. Second, CycleGAN is retrained using these generated images along with clear images obtained through multiscale image decomposition, allowing the model to transform dust-interfered images into clear ones. Finally, color preservation is achieved by selecting the A and B chrominance channels from the small-scale model to maintain the original color characteristics. The experimental results confirmed that the proposed method effectively restores image color and removes sand–dust-related interference, thereby providing enhanced visual quality under sandstorm conditions. Specifically, it outperformed algorithm-based dust removal methods such as Sand-Dust Image Enhancement (SDIE), Chromatic Variance Consistency Gamma and Correction-Based Dehazing (CVCGCBD), and Rank-One Prior (ROP+), as well as machine learning-based methods including Fusion strategy and Two-in-One Low-Visibility Enhancement Network (TOENet), achieving a Blind/Referenceless Image Spatial Quality Evaluator (BRISQUE) score of 17.238, which demonstrates improved perceptual quality, and an Local Phase Coherence-Sharpness Index (LPC-SI) value of 0.973, indicating enhanced sharpness. Both metrics showed superior performance compared to conventional methods. When applied to Closed-Circuit Television (CCTV) systems, the proposed method is expected to mitigate the adverse effects of color distortion and image blurring caused by sand–dust, thereby effectively improving visual clarity in practical surveillance applications.

## 1. Introduction

During yellow sandstorms, which often occur in spring, Mie scatters of red and yellow light occur because the diameter of dust particles in the air is similar to the wavelength of visible light [1]. Images taken during sandstorms often suffer from low contrast and color distortion, resulting in a yellowish tinge. This affects the performance of various tasks using surveillance cameras, common types of conventional sensors, such as traffic monitoring and in-vehicle reversing imaging systems. Therefore, removing the interference caused by dust from images is an important task.

Image-enhancement methods for severe weather conditions have primarily focused on enhancing rain, snow, and fog. However, research on image enhancement for removing sand dust interference is limited and remains an unexplored area. The current method for enhancing images containing yellow-sand interference uses the Atmospheric Scattering Model. Other popular algorithms have been used to enhance images through various color correction methods and image processing schemes to eliminate dust interference, such as the combined Chromatic Variance Consistency (CVC) and Gamma Correction-Based Dehazing (GCD) schemes [2,3]. CVC was used to analyze chromatic variances in the image, thereby generating a transmission map representing the amount of haze in each pixel. The transmission map is then used to recover the original scene radiance. Thereafter, Gamma correction—a nonlinear adjustment of pixel intensity—is applied to defog the image and improve the visibility and overall quality. Although this combined method provides good dust-interference removal from the image, its overall brightness remains insufficient. If the image has a dark area, the image content in the shadow area is not clear, and its details are easily lost.

One sand–dust image enhancement algorithm is the Rank-One Prior (ROP) [4,5]. The ROP framework is an algorithmic approach that enables image restoration under adverse conditions such as haze, sand, and underwater environments. The primary concept behind ROP is to specify environmental conditions by analyzing the scattering map and considering the intensity projection strategy. This method estimates the influence of ambient light through an intensity projection strategy based on the scattering map and performs efficient and rapid scene recovery accordingly. The core concept is to approximate the scattering process using a rank-one structure, thereby simplifying image degradation into an intensity projection problem on a unified spectrum. This approach achieves stable visibility enhancement with low computational complexity, making it suitable for real-time applications such as surveillance cameras and autonomous driving. However, a limitation of the method is that optimal restoration performance requires adjusting hyperparameters according to environmental characteristics.

Dust interference can be eliminated using a fusion strategy that involves machine learning with image-enhancement algorithms [1]. The method combines color correction and a convolutional neural network (CNN). First, a color correction algorithm based on the Gaussian model is used to remove the visual interference caused by dust and balance the hue of the image. The dust removal task is transformed into a haze removal task. Subsequently, the clarity of input images is improved by using a CNN based on the residual structure to eliminate dust-interference nonlinearly. The effect of removing dust interference by yellow sand is not ideal because the overall brightness of the resulting image is low, and its color remains yellowish. In addition, the method based on machine learning requires many datasets, and there is no large benchmark dataset of images captured during sandstorms.

Another CNN-based sand–dust removal module is TOENet. The CCEM (Channel Correlation Extraction Module) of TOENet separates the input image into R, G, and B channels, which are then concatenated to learn the imbalance caused by the weakened B channel and the strengthened R/G channels in sand–dust environments [6]. The concatenated features are processed by an MLP (Multilayer Perceptron) to model the statistical correlations among channels, and the training process computes the loss by comparing the extracted features with the distribution of clean ground-truth images. In addition, perceptual loss is introduced to preserve color components, while skip connection-based multi-scale feature fusion propagates the color correction effect throughout the entire network. However, the output images of TOENet often retain a yellowish tint across the entire scene, indicating that sand–dust is not completely removed. As a result, the images still appear yellowish, and the representation of object details remains weak.

We propose a fusion-based image-enhancement method that integrates Retinex theory with a cycle-consistent generative adversarial network (CycleGAN) [7] to improve the quality of images degraded by yellow-sand–dust interference for a wide range of outdoor environment surveillance cameras. Instead of relying solely on general datasets for training, this approach emphasizes the importance of data preprocessing to guide the learning direction more effectively. By shaping the dataset in a manner that reflects the target image characteristics, the learning outcomes can be better aligned with the desired objectives.

Conventional image-enhancement techniques include gamma correction and histogram equalization. Gamma correction adjusts pixel intensities nonlinearly to improve overall brightness, whereas histogram equalization enhances contrast by redistributing pixel intensity values. However, histogram equalization has inherent limitations, as it can cause excessive contrast and partial detail loss [8].

To overcome these drawbacks, the Recursive Mean-Separate Histogram Equalization (RMSHE) method has been proposed [9]. RMSHE recursively partitions the histogram based on mean values and independently equalizes each sub-histogram, thereby achieving both stable brightness preservation and contrast enhancement. Mathematical analysis has shown that as the recursion depth increases, the mean output brightness converges to the input mean brightness, offering superior brightness preservation and greater flexibility in adjustment compared to conventional methods.

Another approach involves multi-histogram equalization based on the human visual system (HVS) [10]. This method employs logarithmic Michelson contrast measures (AMEs) and entropy-based measures (AMEEs) to automatically select parameters and improve image contrast. However, the goal of this study is to remove the dominant yellow components of sandstorms and haze, which is not well aligned with histogram-based contrast enhancement methods.

Therefore, this study adopts a Retinex-based approach. Nonetheless, many alternative methods to Retinex have also been investigated. For example, the Recursive Rational Filter (RRF) has been used for illumination estimation, leveraging spatial recursivity to realize narrowband low-pass filtering with edge-preserving characteristics [11]. This makes it well suited for real-time and low-complexity implementations. Such an approach represents an alternative to Retinex by employing a different illumination estimator, and it has been reported to achieve stable dynamic range compression without producing excessive halo artifacts. Inspired by these advantages, this study applies Retinex in the frequency domain to efficiently construct training data.

Low- and high-pass filters are also commonly used to process the low- and high-frequency components of images, respectively. Rather than enhancing images directly, these filters serve as auxiliary tools for tasks such as noise reduction and edge detection [12]. Among these techniques, Retinex is a nonlinear image-enhancement method inspired by the human visual system. It enables dynamic range compression and color constancy [13], making it suitable for improving image contrast and clarity while preserving details. Furthermore, applying Retinex in the frequency domain instead of the spatial domain helps reduce the computational complexity and processing time while yielding better results.

In recent years, several survey studies have highlighted the issue of data scarcity in adverse-weather image translation and have reviewed deep-learning techniques designed to operate effectively under limited-data scenarios [14,15]. In studies addressing such data-scarce scenarios, various approaches have been employed to generate datasets required for supervised learning [15].

As no publicly available paired dataset for sand–dust conditions, the proposed method, a CycleGAN framework is employed to generate synthetic training pairs (fake sand–dust images) by creating corresponding sand–dust–affected images from clean counterparts. This approach not only addresses the lack of paired data but also facilitates controlled training for image-to-image translation tasks. The referenced surveys [14,15] provide valuable context by outlining strategies for handling domain gaps and guiding the design of synthetic data generation under sand–dust, thereby reinforcing the motivation and relevance of the proposed method’s dataset construction strategy.

To effectively address the challenges posed by yellow-sand interference, the proposed method consists of the following four key components:

Training 1: A CycleGAN model was trained using unpaired images to generate synthetic paired images that simulate dust interference.

Data Augmentation: Retinex-based processing was applied by transforming clear images from the spatial to the frequency domain via the Fourier transform (FT). Reflection components were extracted by subtracting the illumination layer and enhancing fine details in the clear images.

Training 2: CycleGAN was retrained using a paired dataset composed of Retinex-enhanced clear images and their corresponding synthetic dusty counterparts.

Color Preservation: To maintain high color fidelity, single-scale Retinex (SSR) models with three different sigma values were applied in the LAB color space. The L channel was obtained by combining the outputs from the three scales, whereas the AB channels are taken from the small-scale model to preserve the original color characteristics.

## 2. Materials and Methods

### 2.1. Retinex-Based Tone Processing

Retinex (a combination of Retina and Cortex) is a theory first developed by Edwin Land in 1977. Later, Jobson et al. [16] proposed the two most popular Retinex algorithms, SSR and Multiscale Retinex (MSR). The former can provide dynamic range compression or tonal rendition, depending on the scale (small or large, respectively). The superposition of these two processes is an obvious choice for balancing the two effects. MSR mimics the human visual system. The illumination of objects by incident light is reflected into the imaging system, which ultimately forms the image that we see. The image obtained by the human eye depends on the incident light and its reflection by the surface of the object. The general expression for Retinex is

(1) I(x,y)=R(x,y)∗L(x,y)

where$\;\left(x,y\right)$ is the spatial location, $I\left(x,y\right)\;$is the input image, $R\left(x,y\right)$ is the reflection, and L(x,y) is the illumination by incident light.

Because the signals are continuous and infinite, a computer cannot process them directly. Thus, FT is applied to change the signal from the time domain to the frequency domain, which converts it to a sinusoidal signal with many frequencies and amplitudes that are processable via a computer. However, the calculation burden is exceptionally large because a convolution calculation is used in FT. Hence, fast FT (FFT), which significantly reduces the calculation burden and enables the computer to process the signal, is used instead.

Single-Scale Retinex (SSR) is an image enhancement algorithm derived from Retinex theory, where the reflectance component is estimated by removing the illumination from the input image in the logarithmic domain [17]. This allows suppression of uneven illumination and enhancement of local details. For the i channel of the input images, denoted as $I_{i}\left(x,y\right)$, the SSR output is defined in Equation (2)

(2) $$R_{i}\left(x,y\right)=logI_{i}\left(x,y\right)-\log\left[F\left(x,y\right)*I_{i}\left(x,y\right)\right],\;\;(i=R,G,B\;channel)$$

where $F\left(x,y\right)$ is a Gaussian low-pass filter and * denotes the convolution operation. The Gaussian filter is given by Equation (3)

(3) $$F=\frac{1}{\sqrt{2\pi \sigma^{2}}}exp(\frac{{-r}^{2}}{2\sigma^{2}})$$

with σ representing the Gaussian surround space constant and r is spatial distance in the filter (blur radius).

$R_{i}\left(x,y\right)$ represents the reflectance, obtained by subtracting the log of the estimated illumination (computed via Gaussian smoothing) from the log of the input image [18]. Smaller *σ* values preserve local details and reduce color distortion, whereas larger *σ* values emphasize global illumination correction and tonal balance in the spatial domain.

MSR is based on SSR using multiple sigma values, followed by the weighting of the end results. Compared with SSR, MSR has the advantage of maintaining a high fidelity of the image while achieving color enhancement, local dynamic range compression, and global dynamic compression, but with the disadvantage of haloing. It is defined as

(4) $$R_{MSR}\left(x,y,\sigma \right)=\sum_{k=1}^{n}w_{k}R_{SSR_{K}}(x,y,\sigma_{k})$$

where n is the number of scales, σ = {$\delta_{1},\delta_{2},\ldots ,\delta_{n}$} is a vector of Gaussian blur coefficients,$\;w_{k}$ is the weight associated with the *k*th scale ($\omega_{1}$ + $\omega_{2}$ + … + $\omega_{n}$ = 1), $\left(x,y\right)$ is the spatial location, and $R_{SSR_{K}}\;$is the size of the result after each SSR (*k* times).

**Figure 1:**
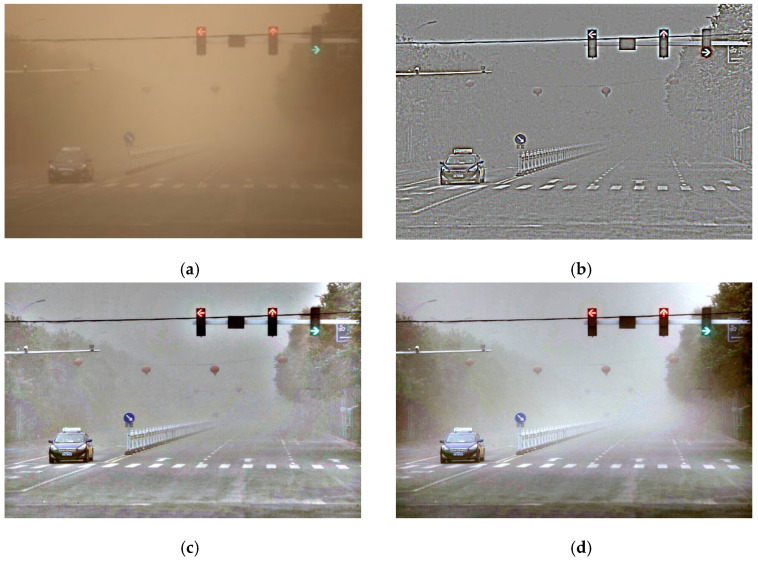
Original image (**a**) and the result of applying single-scale Retinex (SSR) with sigma values of (**b**) 5, (**c**) 40, and (**d**) 150.

Figure 1 illustrates the effect of varying sigma (σ) values in the single-scale Retinex (SSR) algorithm. Specifically, changing the Gaussian kernel parameter σ in Equation (3) modifies the value of $F\left(x,y\right)$ in Equation (2), which alters the illumination component $F\left(x,y\right)*I_{i}\left(x,y\right)$ and generates different reflectance images depending on the sigma scale. In this study, σ = 15, 80, and 250 were used to produce the results shown in Figure 1. This Gaussian kernel–based comparative experiment was as described in [19] to ensure reproducibility.

Figure 1a shows an original image during a sandstorm. After applying an SSR with a sigma value of five (Figure 1b), the color of the image became more normal; however, the local details could be further improved. To achieve this, SSR processing with a sigma value of 40 was performed; however, haloing gradually appeared in the image (Figure 1c). Finally, SSR processing with a sigma value of 150 rendered the image colorless, and the overall details were insufficient (Figure 1d). Hence, small-scale SSR improved the overall tone and contrast of the image, whereas large-scale SSR provided improved local contrast and image details.

### 2.2. CycleGAN-Based Image Translation

Generative adversarial networks (GANs) [20,21] have achieved impressive results in image generation [22,23], image editing [24], and representation learning [25,26]. A basic GAN has a unique structure wherein two neural networks—the generator and the discriminator—compete [27]. The generator is used to generate samples, whereas the discriminator is used to determine whether each sample is true. The generator uses random noise to generate fake images, whereas the discriminator performs binary classification training based on both real and fake images. The discriminator generates a score based on the input image, which indicates whether the image generated by the generator is successful, and thereby further trains the latter to generate a better image. Based on Ian Goodfellow’s definition of GAN [20], they completed the optimization task in the following manner:

(5) $$\underset{G}{\min}\underset{D}{\max}V(D,G)=E_{P_{data}}\left(a\right)[\log D(a)]+E_{p_{z}}(z)[(\log\left(1-D\left(G\left(z\right)\right)\right]$$

where G is the generator, D is the discriminator, V is a value function representing the discriminative performance of the discriminator (the larger the value, the better the performance), $P_{data}(a)$ represents the real data distribution, $P_{Z}(z)$ represents the input data distribution of the generator, and E is the expectation.

The first term, $E_{P_{data}}\left(a\right)[\log D(a)],$ is constructed based on the loss of the logarithmic function of real data. An ideal situation occurs when the discriminator, D, determines whether a data sample is obtained from a real data distribution. Optimization leads to D(a) = 1 for a real data sample.

The second term, $E_{p_{z}}(z)[(\log\left(1-D\left(G\left(z\right)\right)\right]$, is related to the data generated by the generator. The ideal situation is when the discriminator outputs zero in this scenario. Optimization leads to $D\left(G\left(z\right)\right)$ = 0, where z represents the random noise input to the generator. The discriminator maximizes these two terms.

Because it is an adversarial relationship, optimizing G allows it to deceive the discriminator in the second term, thereby making the discriminator accept the generated data even when $D\left(G\left(z\right)\right)$ ≈ 1. Essentially, the discriminator maximizes the two terms, while the generator minimizes the second term, which results in minimizing the objective function. Because the generator and discriminator are in this adversarial relationship, given a fixed generator, the discriminator is trained to maximize the objective function V(D,G) by correctly classifying real data as real and generated data as fake. Therefore, the discriminator optimization function can be expressed as

(6) $$D^{*}(a)=\frac{P_{data}(a)}{P_{data}(a)+P_{G}(a)}$$

(7) $$P_{data}(a)=P_{G}(a),$$

where $D^{*}(a)$ represents the optimal value for discriminator D and given data point a, $P_{data}(a)$ denotes the probability of a being from the real data distribution, and $P_{G}\left(a\right)$ denotes the probability of a being generated by generator G. This implies that the discrimination result for G tends toward zero, whereas the value of $D^{*}$ tends toward one.

For the optimization of $G^{*}$, when the value of $D^{*}$ is fixed, the condition required to minimize G is given by Equation (7). Intuitively, we can also understand that when the distribution of the generator matches that of the real data, the generator is trained to minimize the value of the objective function V(D,G), whereas the discriminator is trained to maximize it, forming a minimax optimization problem.

As its name suggests, the network in CycleGAN (a new type of GAN proposed by the Berkeley Artificial Intelligence Research Lab) is a circle [3]. It addresses the problem of inconsistent image-to-image translation without sufficient paired-image datasets by adding a cycle-consistency loss that feeds back the generated image through an inverse function to ensure transfer parity with its source image. The formula for cycle-consistency loss is

(8) Cycle Loss=∥F(G(a))−a∥+∥G(F(b))−b∥

where ‖.‖ is the pixel-level difference between two images, a is the input image belonging to the *A* domain, b is the input image belonging to the *B* domain, G(a)  and F(b) comprise the generated image, and $F\left(G\left(a\right)\right)$ and G(F(b)) comprise the cyclically reconstructed image from the original image. Unlike Pix2Pix, CycleGAN does not require pairs of images. Moreover, the loss function ensures that converting the image from *Domain A* to *Domain B*, and then vice versa, can be used to reconstruct the original image.

**Figure 2:**
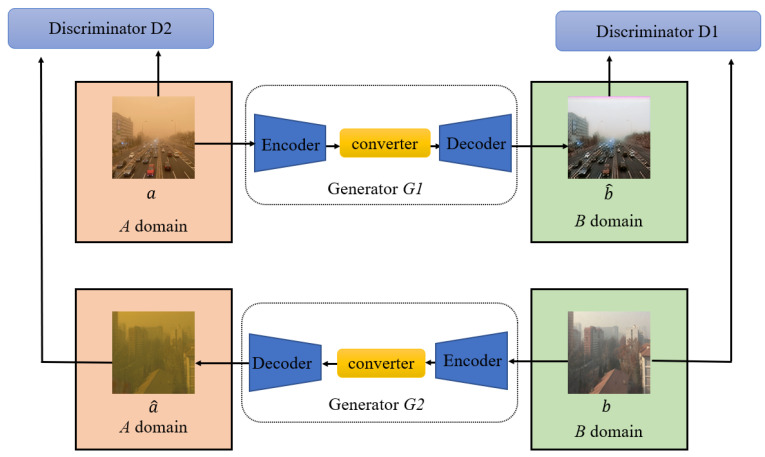
Block diagram of cycle-consistent generative adversarial network (CycleGAN).

As shown in Figure 2, generator *G1* converts the image with yellow sand–dust interference from Domain *A* to Domain *B* and generates a fake clear image, $\hat{b}$, identified by the discriminator as either 1 (true) or 0 (false). The difference between images a and $\hat{b}$ is the GAN loss. After converting $\hat{b}$ in generator *G2*, the clear fake image, $\hat{b}$, in Domain *B* is converted to image $\hat{a}$with yellow sand–dust interference in Domain *A*. The difference between images a and $\hat{a}$ is the cycle-consistency loss. When the clear real image b is input into *G1*, the generated image$\;\hat{b}$ should be as close as possible to input b. The difference between b and $\;\hat{b}$ is called identity loss.

In summary, the basic concept of GANs is to augment the encoder–decoder-based generator with a separate discriminator network that determines whether the generated images are real or fake, and to use its output as part of the loss function. The generator is trained to deceive the discriminator by producing results that appear realistic, which is the key advantage that distinguishes GANs from other deep learning–based generative models.

**Figure 3:**
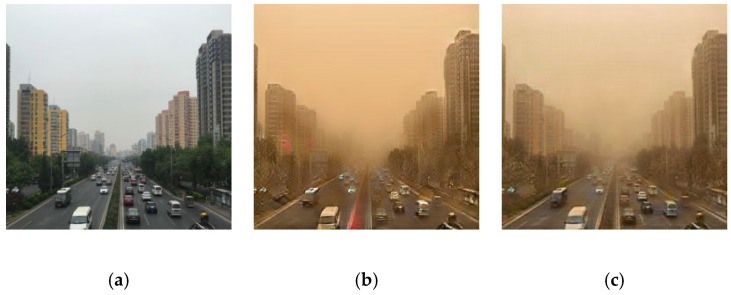
Generation of synthetic stand-dust images using CycleGAN: (**a**) real clean image, (**b**) fake sand-dust image generated by unpaired dataset, and (**c**) fake sand-dust image generated by paired dataset.

**Figure 4:**
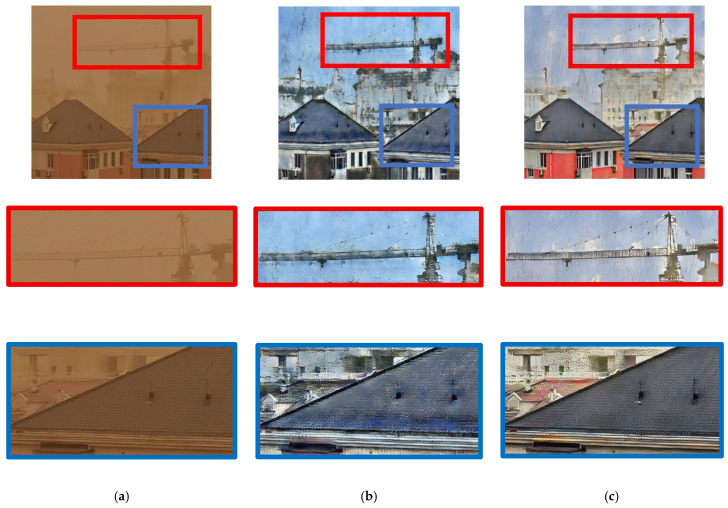
Image translation of sand-dust images into clean images using CycleGAN: (**a**) real sand-dust image, (**b**) clean image generated by unpaired dataset, and (**c**) clean image generated by paired data.

CycleGAN is designed to use unpaired data, and its generator objective function leverages the discriminator adversarial loss to drive the generated samples toward the distribution of the target domain. However, the primary advantage of training CycleGAN with paired data is its ability to leverage paired information to guide the learning process. The objective function of the generator directly includes the mapping error term. Paired data provide explicit pixel-level correspondence between images from different domains, which means that the model can directly learn the mapping between two domains and the pixel-level correspondence. This approach can better capture image features and reduce ambiguity and uncertainty in the drawing process, resulting in more visually appealing and accurate transitions between domains. The results of using CycleGAN for clear and sandstorm-obscured images are shown in Figure 3. The real clear, unpaired training-generated, and paired training-generated images are shown in Figure 3a–c, respectively. The fake sandstorm-obscured image has the same characteristics as the real image with yellow-sand interference, that is, it has a yellowish color and is blurred. The result of applying CycleGAN to sandstorm-obscured and clear images of a tower crane in Figure 4 shows that CycleGAN with paired data is better at removing the yellow-sand interference and has increased image details.

### 2.3. Proposed Method

Since Retinex has the property of removing background components while enhancing image sharpness and brightness, this study employed SSR to guide the learning direction. It was hypothesized that this approach could suppress the global yellow cast and background effects caused by sandstorms while improving image details. In addition, the characteristics of SSR vary with the sigma scale in the frequency domain: a larger sigma results in a narrower frequency response that suppresses high-frequency components and emphasizes low-frequency information, whereas a smaller sigma produces a wider frequency response that allows more high-frequency components to pass through, thereby preserving details with less color distortion. To exploit these properties, three sigma scales were individually trained to generate independent modules, and their outputs were then fused to incorporate the advantages of MSR (Multi-Scale Retinex). For color preservation, the AB channels obtained from the small sigma (1-scale) were used to maintain natural color representation, while the L channel was derived by combining the outputs of the three scales and processed in the LAB space to ensure both detail enhancement and color fidelity.

**Figure 5:**
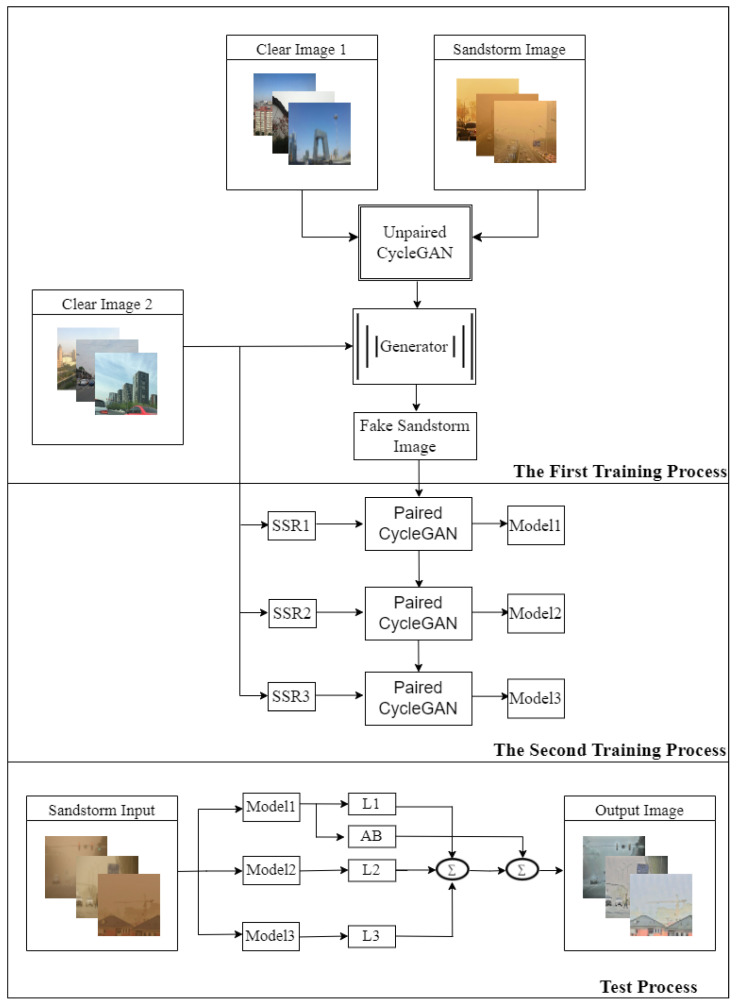
Flow diagram of the proposed algorithm.

The proposed method is divided into three stages, as shown in Figure 5. The first training step involved the generation of fake dust-interference images. This stage was conducted using a dataset consisting of 2000 clear images (referred to as Clear Image 1) and 250 real sandstorm images. Because CycleGAN learning with unpaired data requires a substantial amount of authentic sandstorm-obscured images, their scarcity in real life hampers its progress. Consequently, our approach employed CycleGAN with unpaired data for the initial learning process and runs the generator in the “clear to sandstorm” direction. This initial learning phase provided a substantial dataset of paired images to facilitate subsequent learning steps.

The second training step involved training CycleGAN with paired enhanced clear images. In this stage, another set of 2000 real images (referred to as Clear Image 2) was enhanced using the Retinex algorithm at three different scales. These enhanced images were then paired with 2000 synthetic sandstorm images generated in the first training step to form paired training data. Three models were obtained by applying Retinex enhancement to the dataset images at three different scales. Each resulting dataset exhibited distinctive characteristics, and this preprocessing step enriched the visual content, providing improved input data for the second phase of CycleGAN training.

In the test process step, after obtaining modules and their respective characteristics, the processing was performed on the L channel and then the combined A and B channels of the SSR model for favorable color preservation.

#### 2.3.1. First Training Process: Fake Dust-Interference Image Training

This study proposes a method to enrich the paired dataset by initially training unpaired data and then using the trained model to generate paired data for subsequent training. Because paired data have a clear mapping relationship based on the comparison between paired and unpaired images, paired training can lead to high-quality translation as well as faster convergence, shorter learning time, and better control characteristics. As shown in Figure 4, after training CycleGAN with both paired and unpaired data, the model was applied to the enlarged region highlighted by the blue box. The results indicated that training with paired data achieved superior dust removal and produced a more visually consistent clean image.

**Figure 6:**
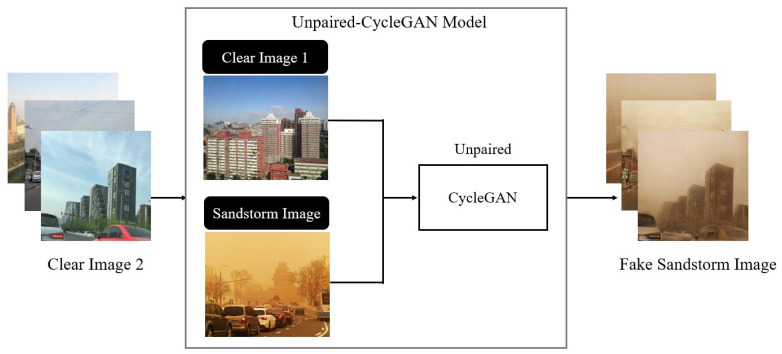
Schematic of fake dust-interference image training.

Although training CycleGAN with paired datasets yields better results, real-world image datasets obscured by yellow sand are lacking and paired datasets are almost nonexistent. However, synthetic paired data can be generated by translating images from one domain to another using an unpaired CycleGAN model. This method not only expands the dataset, reduces the dependence on limited paired data, and provides additional training samples for subsequent supervised learning tasks, but is also flexible and adaptable. We can control the distribution of the generated pairs, tailor it to a specific need, or create scenarios that are difficult to obtain from paired real-world data. It is important to note that the quality and validity of the generated paired data depend heavily on the performance and capability of the initial unpaired CycleGAN model. To ensure that the generated image pairs are of sufficient quality and preserve the desired characteristics of the target domain, careful consideration must be given during data generation. Therefore, we first trained CycleGAN using unpaired images and then used the trained model to generate paired data, referred to as “fake dust-interference image training,” as illustrated in Figure 6.

#### 2.3.2. Second Training Process: Paired Training CycleGAN with Enhanced Clear Images

To enhance the performance of CycleGAN, the proposed method first augmented the dataset and then performed paired training. This involves using Retinex to enhance the data before training CycleGAN with paired data. Images were transformed from the spatial to the frequency domain before being processed using Retinex. In the LAB color space, the L channel provides luminance information, whereas the A and B channels provide color information. The LAB color space exhibits excellent adaptability to changes in illumination conditions. The luminance information on the L channel experiences minimal variation under different lighting conditions. In addition, the color uniformity is high, implying that during color preservation, the variation in color across different regions is smoother. In contrast to the relatively complex relationships between the color components in the RGB color space, which can lead to unexpected effects during color preservation, working in the LAB color space enables the separation of color image luminance. This ensures that the color information remains unaffected, thereby preserving the color fidelity.

**Figure 7:**
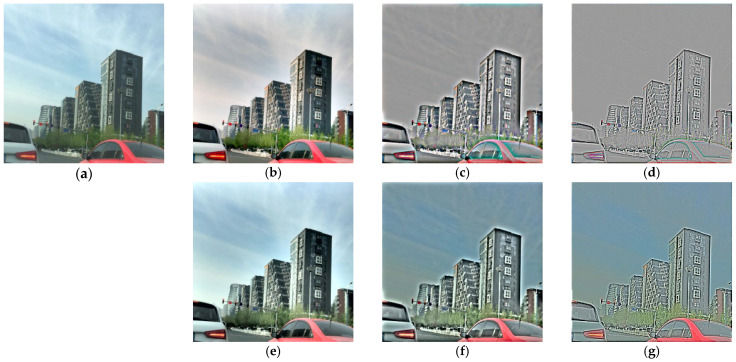
Comparison of color-processed images: (**a**) the original clear image, (**b**–**d**) SSR-enhanced results in the RGB color space using sigma values of 1, 10, and 40, respectively, and (**e**–**g**) SSR-enhanced results in the LAB color space using the same sigma values.

Part of the car in Figure 7 has color distortion, which is attributed to the Retinex enhancement process. During Retinex enhancement, luminance information is accentuated, while color information might undergo compression or alteration. Adjustments in brightness and contrast applied to individual color channels can disrupt the balance between them, leading to color distortion or loss and reducing the authenticity of image colors.

Retinex-based enhancement technology can significantly improve the visual quality of an image by enhancing brightness, contrast, color, fine details, textures, and edges, which ensures that the output image has improved visual features compared to the input image. Augmenting a dataset with Retinex better preserves these details during CycleGAN training with paired images, which can lead to more accurate and better preserved translations that retain important visual features. Retinex-based enhancement can also reduce noise and artifacts in images, resulting in clearer and smoother data. Training CycleGAN on a paired-image dataset with reduced noise and artifacts can help the model focus on learning the desired mapping between domains rather than being affected by undesirable artifacts. In addition, augmentation by Retinex can enhance uniformity and consistency, thereby improving training convergence. The enhanced image provides a more stable and reliable training signal, which can help the CycleGAN model trained on paired images to converge faster and more efficiently.

**Figure 8:**
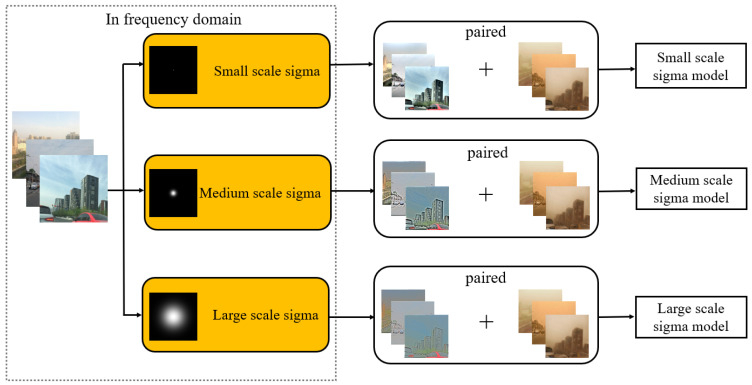
CycleGAN training with Retinex-enhanced images.

As shown in Figure 8, CycleGAN training with Retinex-enhanced images involves transferring clear images from the spatial domain to the frequency domain and then performing SSR preprocessing in the LAB color space. The reason for moving to the frequency domain is that image processing therein can significantly reduce the computing time. Thus, the L channel in the LAB color space was used for data augmentation.

**Figure 9:**
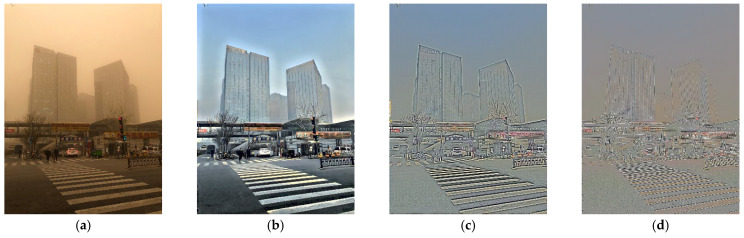
CycleGAN training with Retinex-enhanced images: (**a**) original sandstorm-obscured image, (**b**) small-scale mode, (**c**) medium-scale mode, and (**d**) large-scale model.

The small-scale model (Model 1) can better preserve and transfer the color features of the image while enhancing both the overall detail and contrast. However, the drawbacks are also evident in Figure 9b, with a halo appearing in the sky portion. While small-scale Retinex enhances image details, it tends to generate noise and artifacts that can be propagated or amplified during CycleGAN learning.

The medium-scale model (Model 2) is capable of better adjusting the image texture and details. In addition, it can appropriately enhance the image contrast, thereby making variations in brightness more pronounced and boosting the overall visual effect. Drawbacks include focusing on local details that can disrupt the overall balance of the image, which affects its natural feel. Furthermore, inadequate color fidelity is another disadvantage, which is evident in the building sign portion of the image in Figure 9c.

A large-scale model (Model 3) can accentuate the features and structures within the image, thereby enhancing the sense of depth. It also adjusts the image contrast, which renders the edge texture clearer and emphasizes local variations in brightness, as shown in Figure 9d. However, processing datasets in the frequency domain can result in the loss of local details. This can lead to a large-scale model potentially losing the ability to enhance certain finer details during learning. Furthermore, inadequate color fidelity is an unavoidable concern associated with this approach. Therefore, careful selection of an appropriate large-scale Retinex preprocessing model is necessary.

To consider the local and overall details, the experimental results for the large-, medium-, and small-scale models were combined, and a weight of 1/3 was used to obtain the final image. Compared with the previous image, the processed image has dynamic compression and shows details in the shadows, while the local details and overall details were greatly improved.

#### 2.3.3. Image Preservation

**Figure 10:**
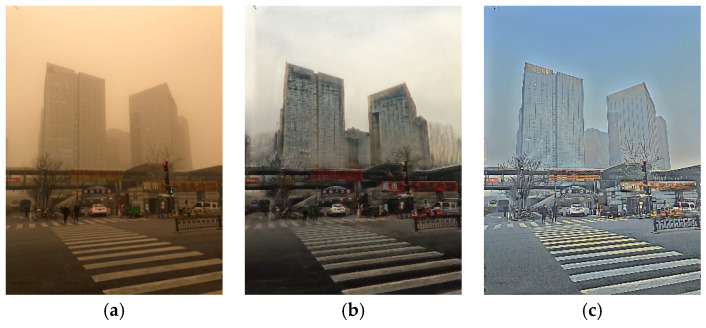
Results of CycleGAN-based image translation: (**a**) the input image obscured by a sandstorm, (**b**) the output from a CycleGAN trained with unpaired data, and (**c**) the output from a CycleGAN trained with the proposed method.

The composite results of the three SSR models at different scales in the RGB channel still created serious color deviation; therefore, it is necessary to reduce color deviation through color preservation. As shown in Figure 5, after transferring the image to the LAB space, the small-scale sigma model (Model 1) provided normal color but poor local details, whereas the large-scale sigma model (Model 3) provided poor color but excellent local details and contrast. Putting the image obtained via the three SSR models with a weight of 1/3 through the L channel and the color parts through the A and B channels of the LAB color space using the small sigma SSR model, and then transferring the result to the RGB color space, effectively reduced the color deviation and maintained excellent contrast and details, as shown in Figure 10.

## 3. Simulation Results

To validate the proposed system, we used Visual Studio Version:1.73.0 to test the proposed method by reading the images using Python 3.8, PyTorch 1.8.1, and a hardware configuration comprising an Intel core i7-10700K and a CPU (Santa Clara, CA, USA) running at 3.79 GHz and an NVIDIA GPU (GeForce RTX 3080) (Santa Clara, CA, USA). To generate synthetic sand–dust images for unpaired training, 2000 real clear images and 200 real sandstorm images were used. For SSR-based paired training, 2000 real clear images and their corresponding 2000 synthetic sand–dust counterparts were employed. Input images were resized to 286 × 286 and randomly cropped to 256 × 256.

The generator was designed as a ResNet with nine residual blocks, and the discriminator was a 70 × 70 PatchGAN with three convolutional layers. Both networks used 64 feature maps in the first layer. Training was performed for 200 epochs, with the Adam optimizer (learning rate = 0.0002, β1 = 0.5). The loss function was the least squares GAN loss (LSGAN), combined with cycle-consistency losses (λA = 10, λB = 10) and an identity loss weighted by 0.5. Instance normalization was applied, and dropout was not used during training.

To verify the dust interference removal effectiveness of the proposed image enhancement method, experiments were conducted and an ablation table was constructed. The ablation study compared three approaches: unpaired CycleGAN, paired CycleGAN using fake sand–dust images, and the proposed 3-scales SSR CycleGAN method. Finally, the performance of the proposed method was comprehensively evaluated against existing dust removal techniques.

The ablation study in Table 1 demonstrates the progressive improvements from the CycleGAN to the proposed method. The unpaired CycleGAN provides moderate quality, while the paired CycleGAN trained with fake sand–dust images shows no improvement in most metrics, except for the LPC-SI sharpness metric. In contrast, the proposed 3-Scales SSR CycleGAN achieves the lowest BRISQUE, the highest MCMA, and significantly better sharpness scores (S3 and LPC-SI), clearly demonstrating the advantage of the proposed method.

Table 1 demonstrates that as the SSR scale increases, image details become more pronounced, which is evident from the image-sharpening evaluation. However, larger SSR scales tend to degrade the BRISQUE score and yield limited improvements in contrast, as indicated by the image quality evaluation. Interestingly, when multiple SSR scales are combined, both image quality and sharpness metrics show improvement. This suggests that, rather than relying on a single SSR scale for sand–dust removal in image transformation, employing a representative combination of SSR scales provides greater enhancement. The sigma values for SSR scales were determined experimentally in the frequency domain.

### 3.1. Comparison with Conventional Methods

**Figure 11:**
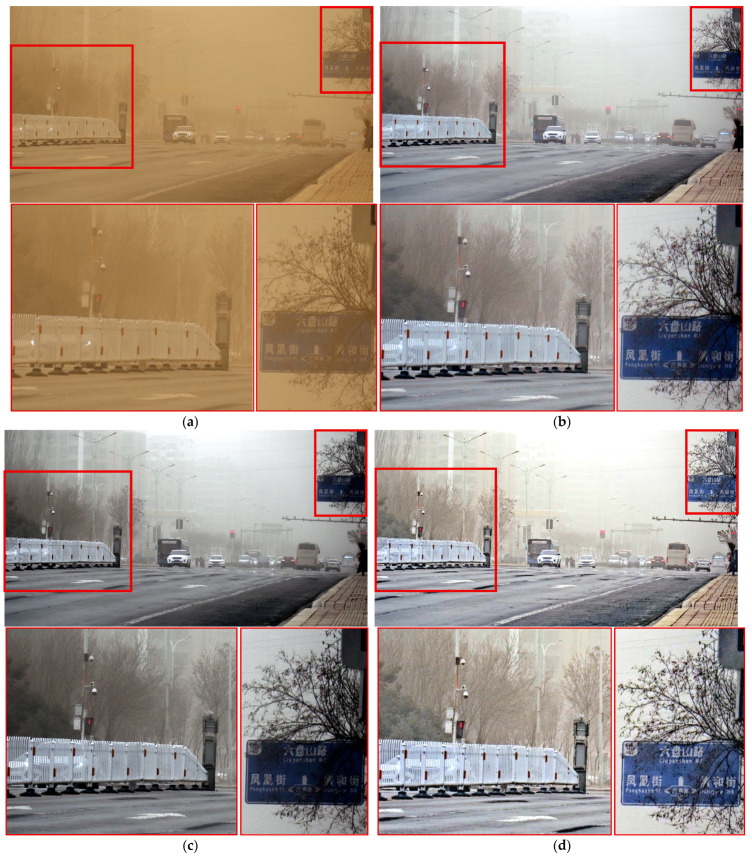

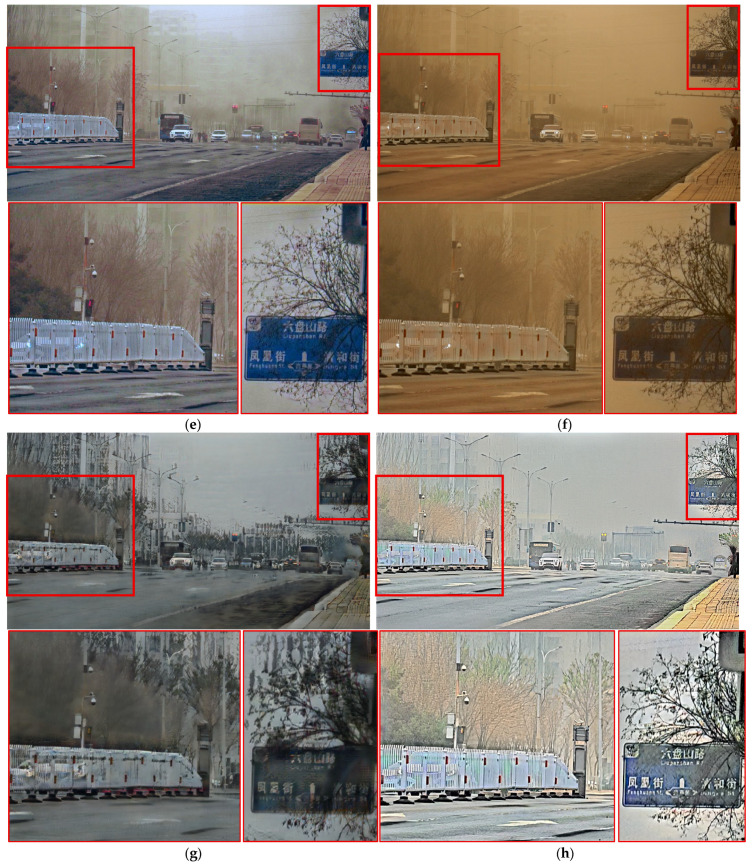
Comparison of enhancement methods on a road scene with traffic signs under sand-dust condition: (**a**) the original image, (**b**) SDIE, (**c**) CVCGCD, (**d**) ROP+, (**e**) Fusion strategy, (**f**) TOENet, (**g**) CycleGAN (unpaired), and (**h**) the proposed method.

**Figure 12:**
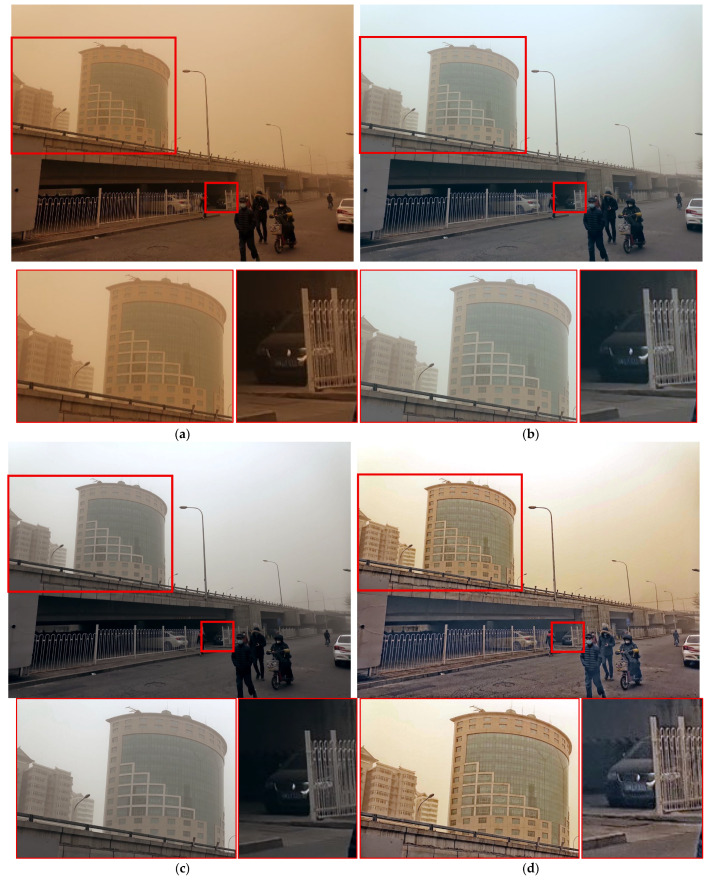

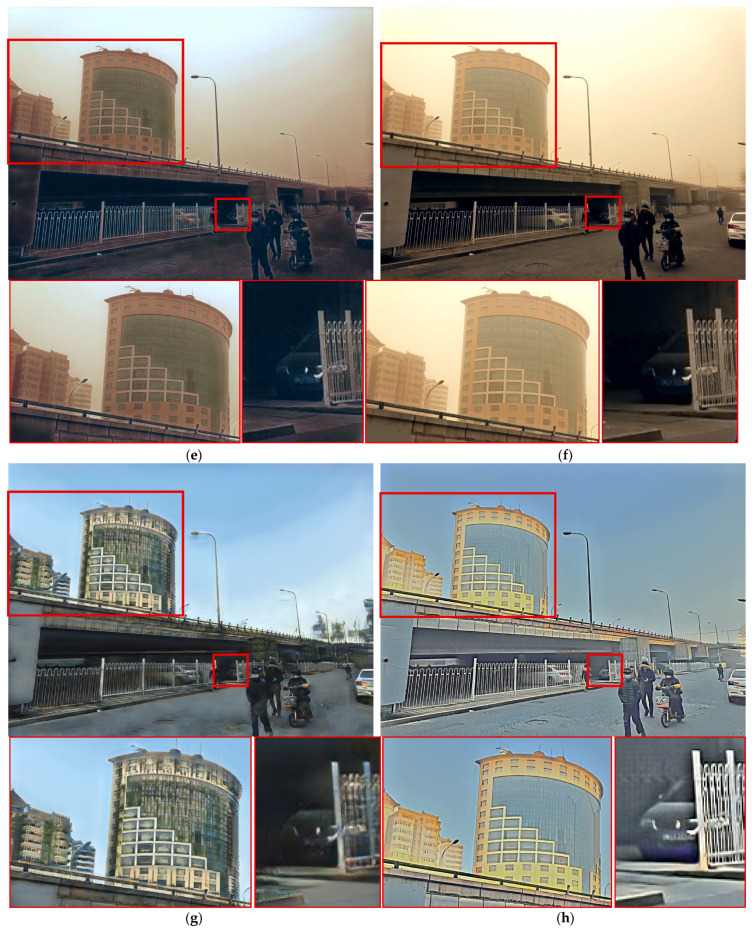
Comparison of enhancement methods on buildings and an underpass by sand-dust: (**a**) the original image, (**b**) SDIE, (**c**) CVCGCD, (**d**) ROP+, (**e**) Fusion strategy, (**f**) TOENet, (**g**) CycleGAN (unpaired), and (**h**) the proposed method.

**Figure 13:**
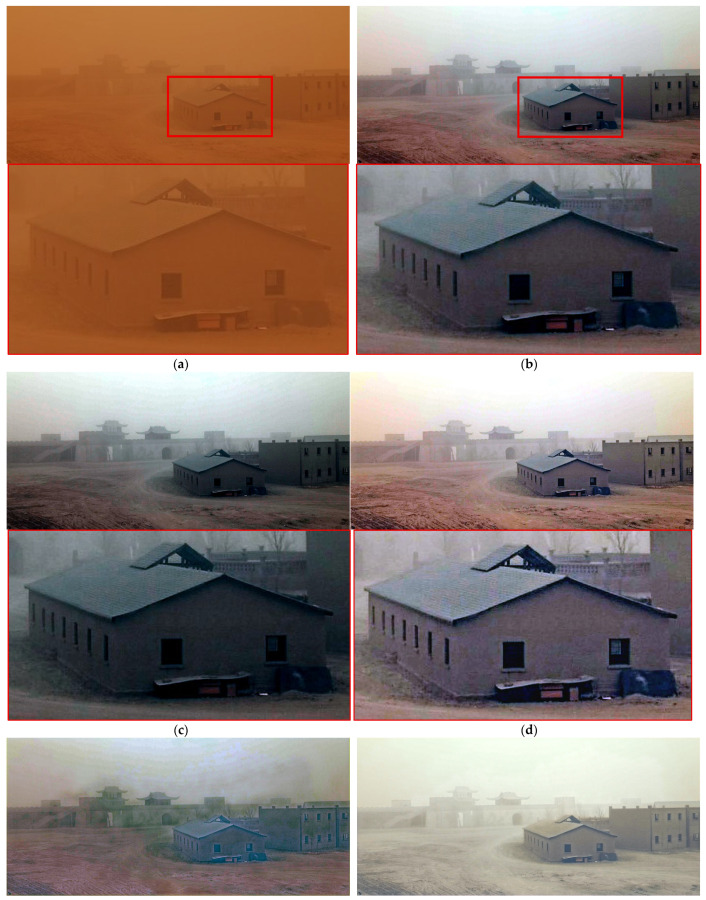

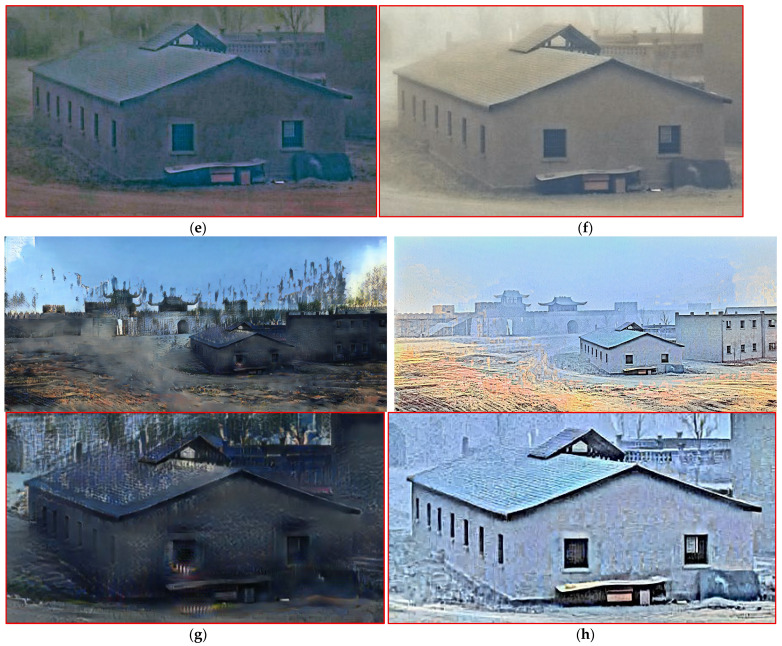
Comparison of enhancement methods on the desert environment containing buildings degraded by sandstorms: (**a**) the original image, (**b**) SDIE, (**c**) CVCGCD, (**d**) ROP+, (**e**) Fusion strategy, (**f**) TOENet, (**g**) CycleGAN (unpaired), and (**h**) the proposed method.

**Figure 14:**
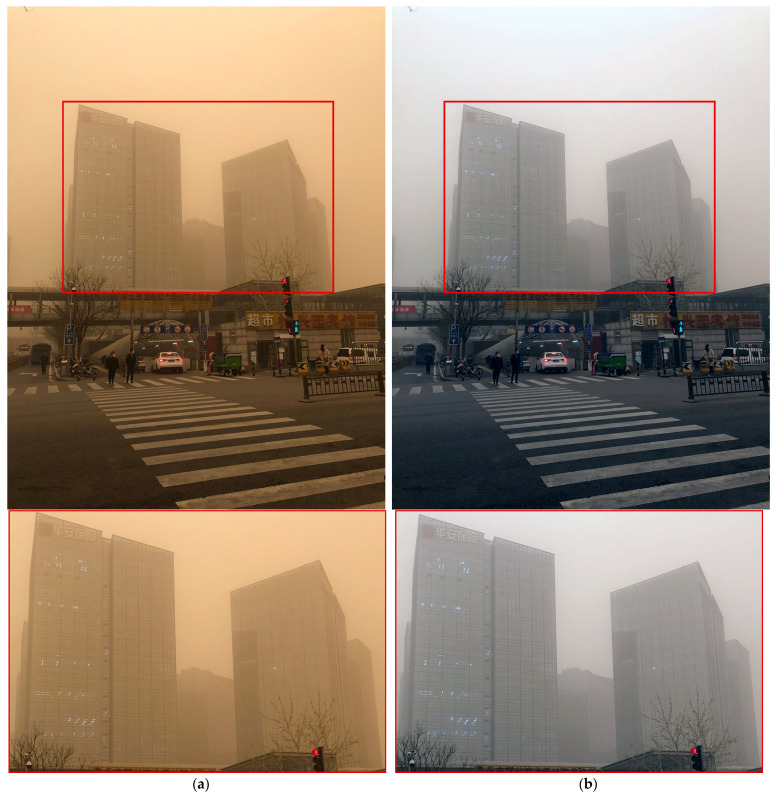

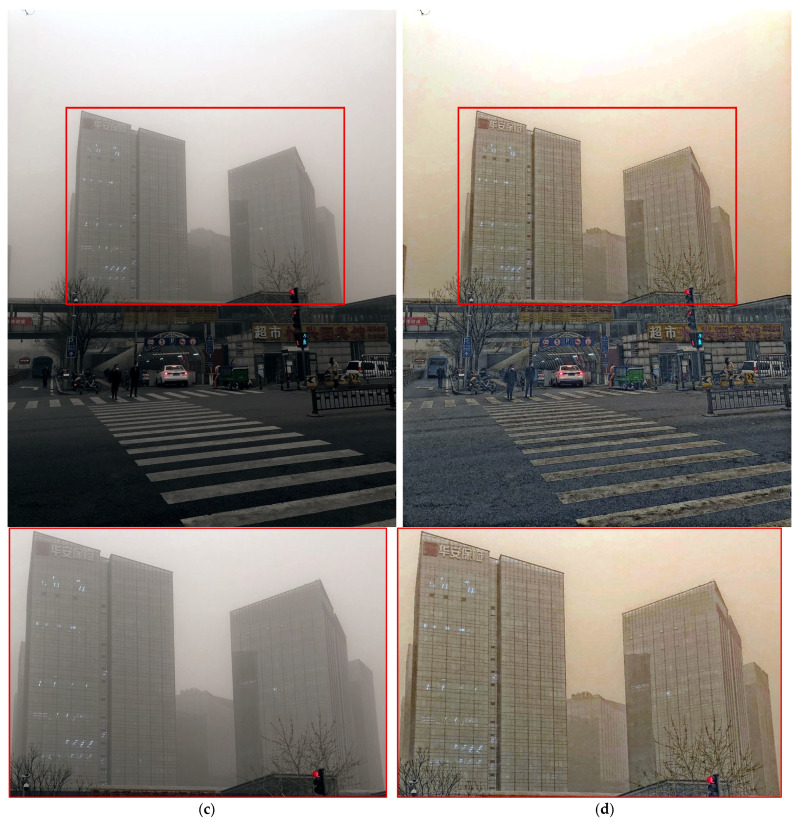

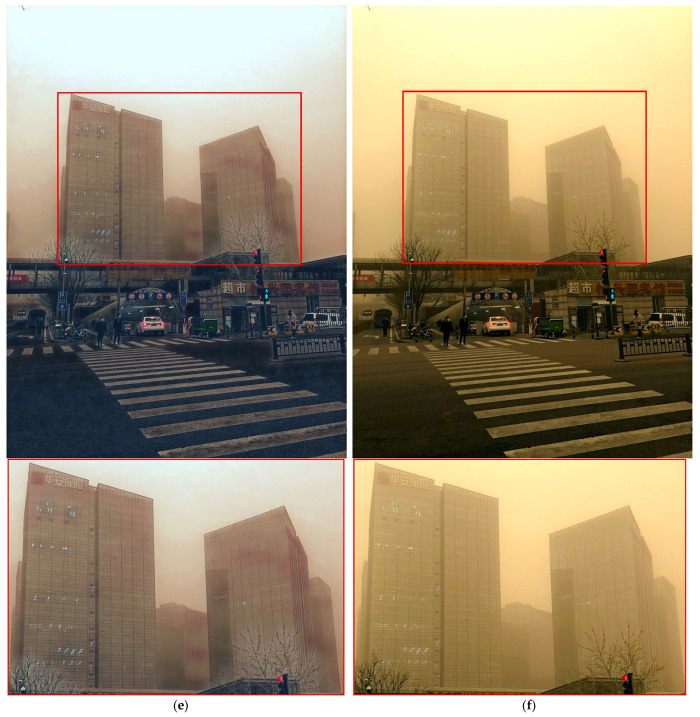

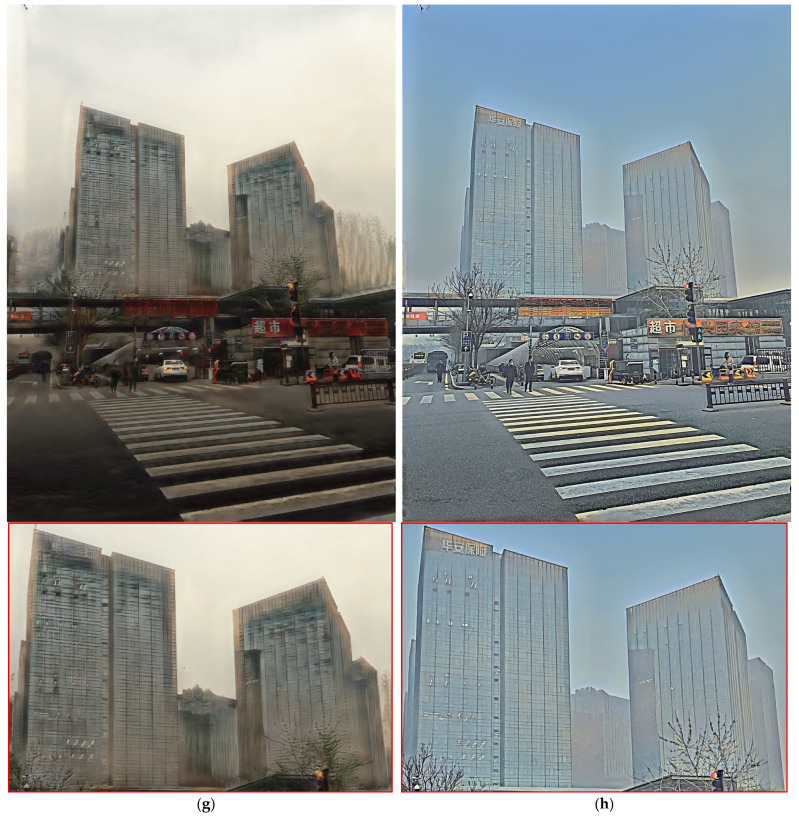
Comparison of enhancement methods on a crosswalk scene with large buildings affected by sand-dust: (**a**) the original image, (**b**) SDIE, (**c**) CVCGCD, (**d**) ROP+, (**e**) Fusion strategy, (**f**) TOENet, (**g**) CycleGAN (unpaired), and (**h**) the proposed method.

**Figure 15:**
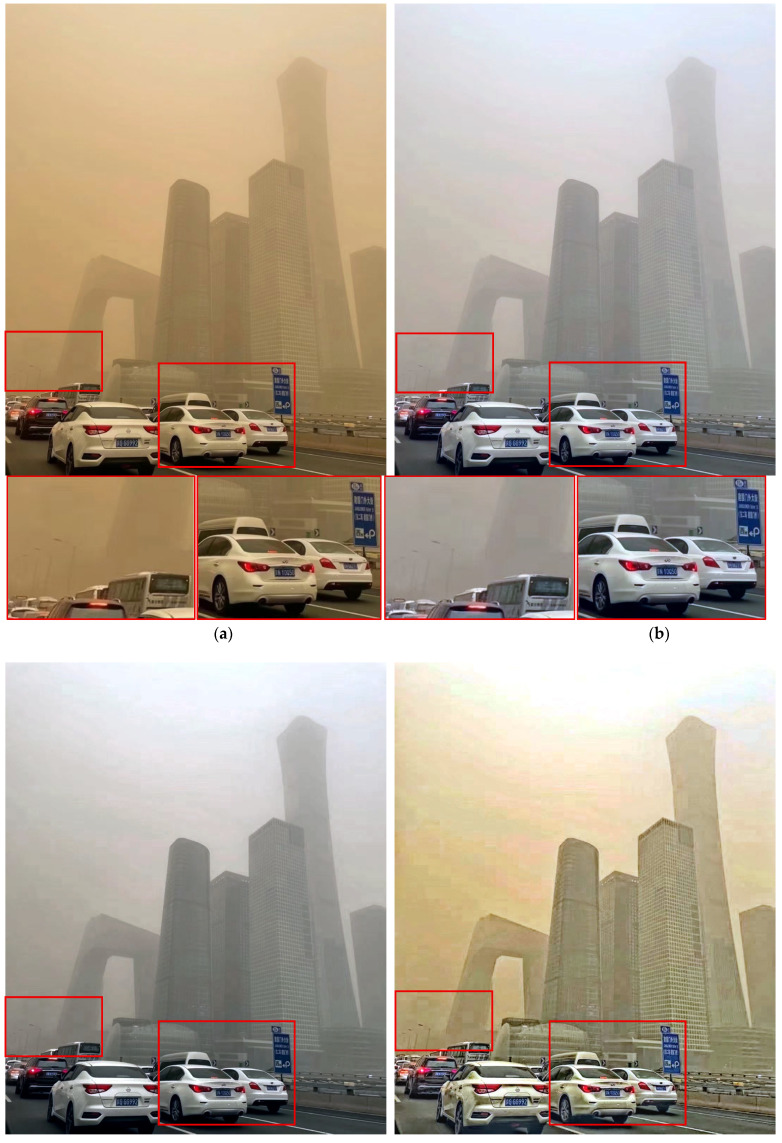

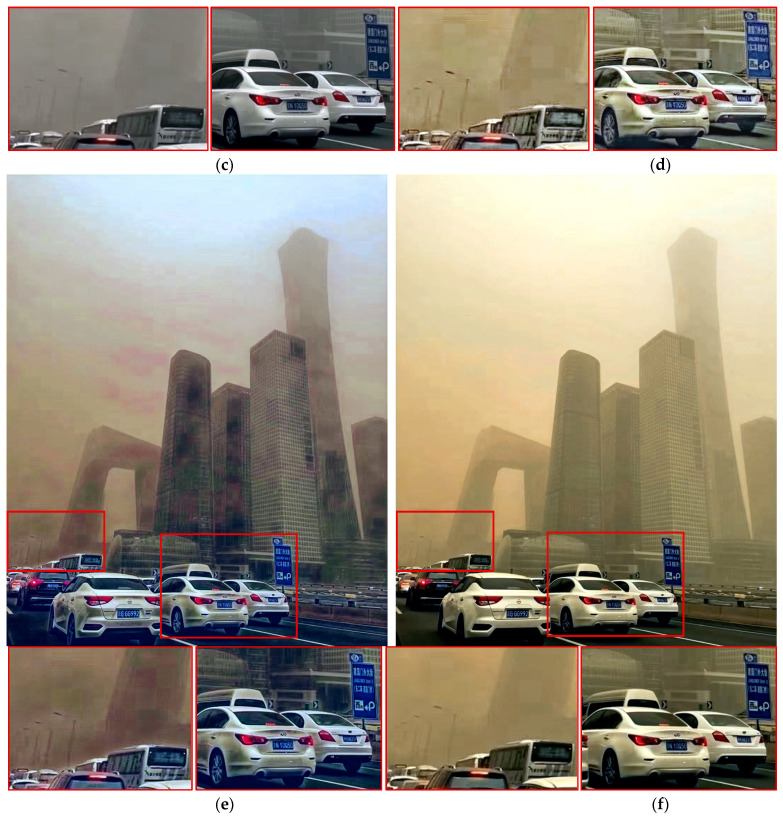

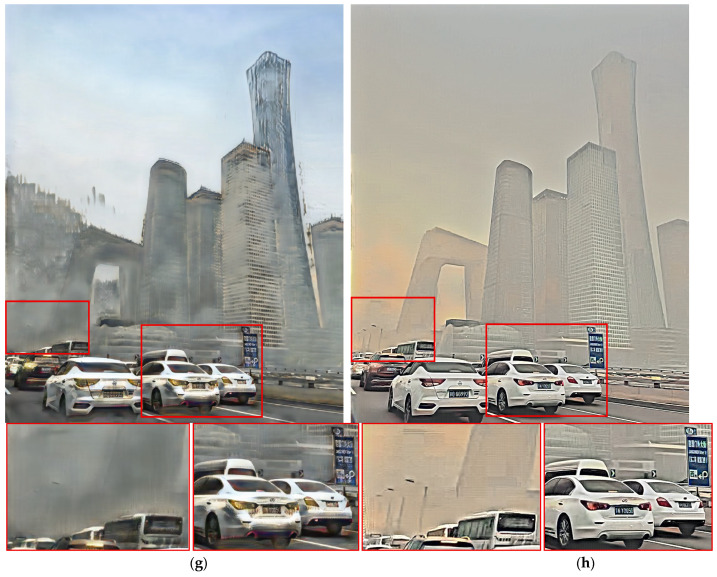
Comparison of enhancement methods on a congested roadway with traffic signs and surrounding buildings: (**a**) the original image, (**b**) SDIE, (**c**) CVCGCD, (**d**) ROP+, (**e**) Fusion strategy, (**f**) TOENet, (**g**) CycleGAN (unpaired), and (**h**) the proposed method.

**Figure 16:**
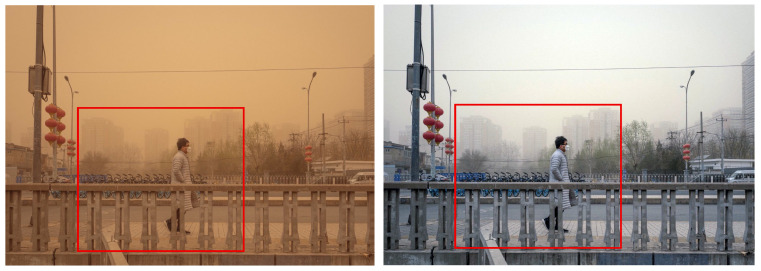

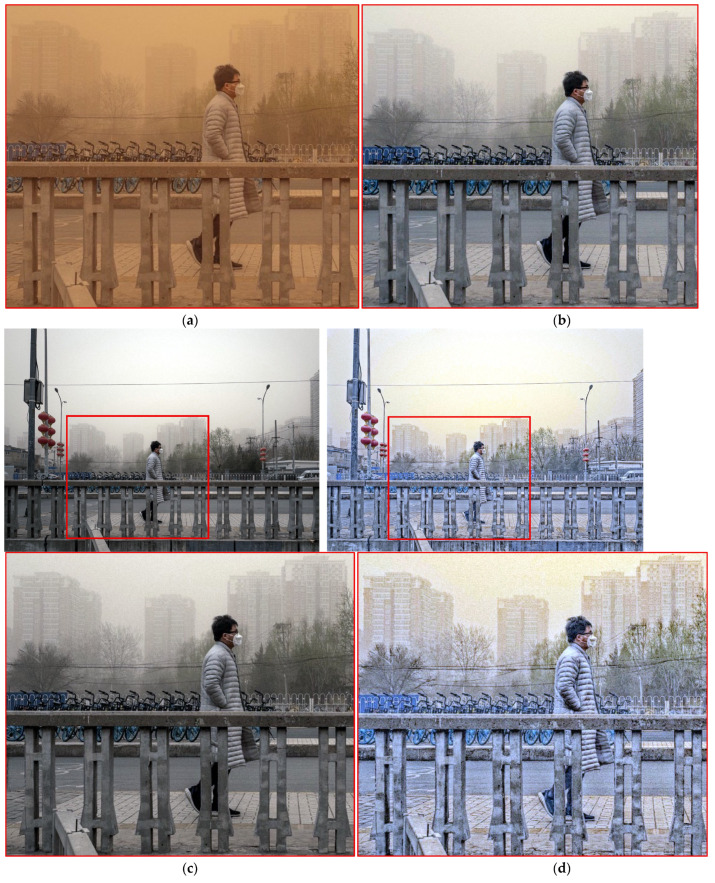

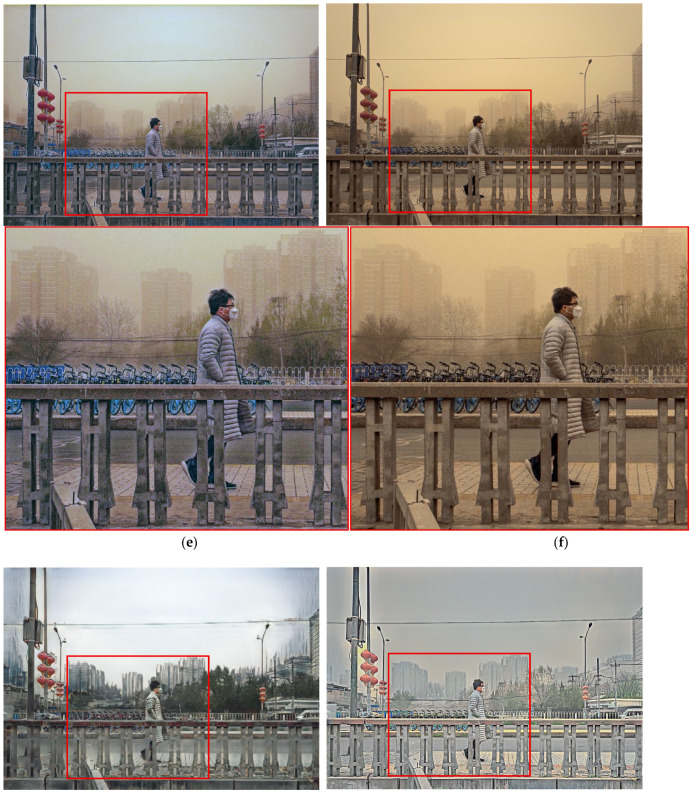

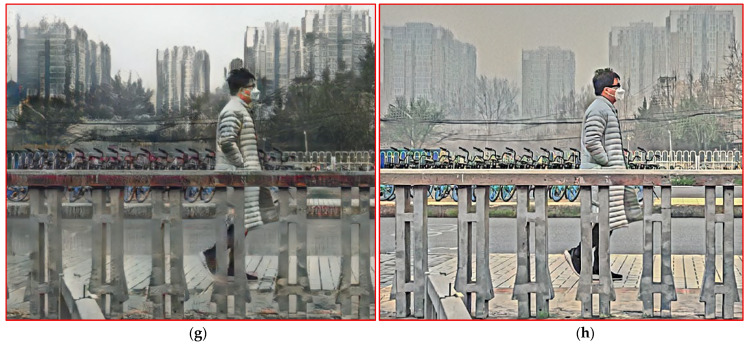
Comparison of enhancement methods on a bridge scene with a pedestrian and background buildings degraded by sand-dust: (**a**) the original image, (**b**) SDIE, (**c**) CVCGCD, (**d**) ROP+, (**e**) Fusion strategy, (**f**) TOENet, (**g**) CycleGAN (unpaired), and (**h**) the proposed method.

Figure 11, Figure 12, Figure 13, Figure 14, Figure 15 and Figure 16 present a comparison of the sand–dust removal performance between the proposed method and conventional methods. In Figure 11, most methods, except TOENet, effectively removed the yellowish background components caused by sand–dust. Among them, ROP+ and the proposed method most successfully eliminated sand and haze while preserving object details. Although ROP+ showed more natural and superior color representation, the proposed method achieved the best text clarity on the signboard among all results.

In Figure 12, ROP+, the Fusion strategy, and TOENet all failed to completely remove the yellowish background components. Compared with SDIE and Chromatic Variance Consistency and Gamma Correction-Based Dehazing (CVCGCD), the proposed method most clearly preserved the contrast of the car parked under the bridge.

Figure 13 evaluates performance under severe desert sandstorm conditions. While all methods effectively removed sandstorm components, ROP+ and TOENet provided slightly superior color representation compared to the proposed method. However, the proposed method outperformed others in preserving fine details, such as small windows, minor structures behind the main building, and ground textures.

Figure 14 shows a comparison in road-side environments. SDIE, CVCGCD, and the proposed method all removed the yellowish components effectively. However, SDIE and CVCGCD failed to sufficiently remove the haze-like elements, resulting in inferior building detail compared with the proposed method. ROP+ preserved details in some areas at a level similar to or better than the proposed method, but it did not fully remove the yellowish sand components. Furthermore, its road, crosswalk, and building contrast was lower, and excessive sharpening artifacts were observed.

Figure 15 compares performance in road-driving scenarios. All methods except the proposed one exhibited noticeable ripple(wave)-like noise. In contrast, the proposed method preserved the details of the white car and especially the blue signboard most clearly, confirming that it is the most suitable for driving environments.

Finally, Figure 16 shows that the Fusion strategy and TOENet failed to completely remove the yellowish components. For distant buildings, both ROP+ and the proposed method preserved details effectively. However, the difference was clear in person representation. The proposed method depicted clothing edges without black noise artifacts, whereas the ROP+ results exhibited distinct black noise on the collar, reducing the fidelity of person representation.

In summary, CycleGAN provided overall natural colors but suffered from insufficient sharpness and noticeable noise. SDIE and CVCGCD lacked overall brightness and failed to reveal details in dark areas. The Fusion strategy produced satisfactory contrast but retained a yellowish tone, with insufficient sand–dust removal and poor detail recovery in dark regions. ROP+ achieved detail expression and color representation comparable to or better than the proposed method in some cases but failed to remove yellowish sand components in others. By contrast, the proposed method consistently demonstrated superior sandstorm interference removal, adequate overall brightness, clear details in dark regions, and enhanced local detail preservation.

### 3.2. Objective Assessment

The results of each module were objectively compared using four image assessment metrics. To objectively evaluate the performance of the proposed algorithm on the processing of images containing dust interference, we adopted non-reference index evaluation methods perception-based image quality evaluator (PIQE) [28] and blind/referenceless image spatial quality evaluator (BRISQUE) [29] for image quality evaluation, Maximizing Contrast with Minimum Artifact (MCMA) for image contrast metrics [30], and spectral and spatial sharpness (S3) [31] and local phase coherence sharpness index (LPC-SI) [32] for image-sharpening evaluations. PIQE first divides the image into blocks, calculates the activity of the blocks, and calculates the three types of distortions of block effects, blur, and noise for the active jump blocks, and finally accumulates the quality score of the processed image. BRISQUE extracts features based on on-site statistical information from the image to be tested and inputs them into a support vector machine trained to predict the image quality based on these features. Finally, the support vector machine outputs an image quality evaluation score. S3 utilizes both the spectral and spatial properties of an image. For each block, it measures the slope of the magnitude spectrum and total spatial variation. These results were then adjusted to account for visual perception, and the adjusted measures were combined via a weighted geometric mean. LPC-SI is a sharpness measure, where sharpness is identified as a strong local phase coherence evaluated in the complex wavelet transform domain. The algorithm correlates well with subjective quality evaluations. It also detects other image artifacts that can affect perceived clarity, such as compression, median filtering, and noise contamination.

BRISQUE has no closed-form formula. It extracts Natural Scene Statistics (NSS) features from locally normalized luminance coefficients and predicts image quality using a trained SVM regressor.

PIQE also does not have an explicit formula. It divides the image into blocks, computes block-wise distortion measures, and aggregates them with perceptual weights.

MCMA is a contrast optimization metric with artifact suppression. Specifically, PU penalizes excessive local smoothness, HSD penalizes histogram-shape deviations between the input and the enhanced image (using 8-bin rolling aggregation after denoising the histogram tails), and DRO rewards wider effective dynamic-range utilization.

(9) MCMA=(a⋅PU+b⋅HSD+c⋅DRO+1)

where a = −0.7, b = −0.3 and c = 0.4.

S3 estimates sharpness based on structural and spectral information of the image.

(10) $$S3=\left(S_{spectral}^{\alpha }\right)\cdot \left(S_{spatial}^{1-\alpha }\right).\;(0\leq \alpha \leq 1)$$

where $S_{spectral}$ is spectral sharpness, based on the power spectrum slope. $S_{spatial}$ is a spatial sharpness, based on local total variation. α = 0.5, which is commonly used to give equal weight to spectral and spatial sharpness.

LPC-SI Measures local phase coherence, which reflects sharpness and fine structural details.

(11) $$LPC-SI=\frac{\sum_{i=1}^{N}LPC\left(i\right)\cdot u\left(i\right)}{\sum_{i=1}^{N}u\left(i\right)}$$

where $LPC\left(i\right)$ is a local phase coherence value at pixel i.

(12) $$u\left(i\right)=e^{\left(-\frac{i}{N\cdot \beta_{k}}\right)}$$

where N is the number of pixels in the central cropped region, and $\beta_{k}\;$ is controlling the decay speed (commonly set to $1\times 10^{-4}$).

Table 2 compares the metric scores of the different methods. The bold and underlined values indicate the best and second-best performances, respectively. The results in Table 2 indicate that CycleGAN achieved the lowest score in the PIQE evaluation, indicating the best image quality, followed by the proposed method. In the BRISQUE evaluation, the proposed method scored the lowest, whereas in the MCMA, S3 and LPC-SI evaluations, it scored the highest. Therefore, overall, our method outperformed conventional methods.

**Figure 17:**
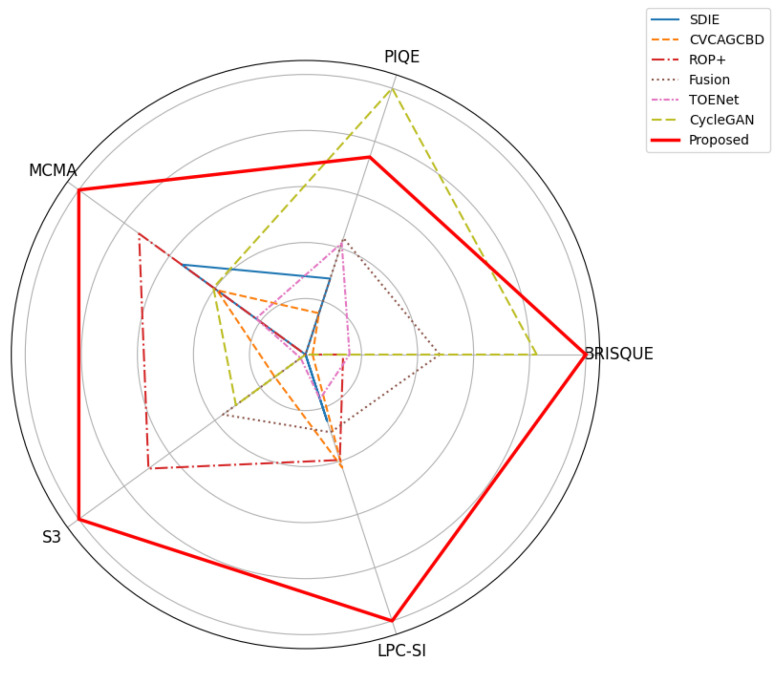
A radar chart for comparative evaluation of overall methods performance based on no-reference metrics.

Figure 17 shows the results of each evaluation method. The radar chart presents a comparative evaluation of the overall performance of the methods based on no-reference image quality metrics. All metric values are normalized to the range [0, 1], with lower-is-better measures (BRISQUE, PIQE) inverted prior to normalization, such that higher values (closer to 1) consistently indicate better performance. This normalization provides a consistent basis for cross-metric comparison and facilitates a comprehensive evaluation of overall performance differences.

Table 3 additionally reports quantitative complexity metrics for prior methods under identical hardware and image-resolution settings, with a particular focus on processing time. As shown, the proposed method exhibits 2.2–4.5 times longer latency than conventional sand–dust removal algorithms (e.g., SDIE, TOENet, etc.). The increased latency primarily is attributable to the accumulation of computations in post-processing and inter-module composition. This reflects a deliberate design decision to suppress artifacts and improve image quality, and thus entails an unavoidable trade-off between quality and computational complexity. To further mitigate latency in practical deployments, dedicated hardware acceleration, for example, via custom IC or FPGA designs optimized for the target operators, will be explored as future work.

## 4. Discussion

This study proposes an image-to-image translation framework for restoring sandstorm-degraded images using CycleGAN trained with SSR-based preprocessing. Three different SSR scales were individually trained to build separate learning modules, and the color information from the 1-scale SSR was incorporated into the final framework. This choice was made because the 1-scale SSR most effectively preserved the natural color distribution while transforming sandstorm images closer to clean ones. However, due to the characteristics of SSR-based training, color desaturation may occur, and in some cases, the generated images may exhibit locally unnatural color representations.

The performance evaluation was conducted on images with a resolution larger than SD (480p) but lower than HD (720p). The generation of CycleGAN-based training images required approximately 1.294 s per frame, with an additional 0.21 s for post-processing, including fusion and color information transfer, resulting in a total processing time of about 1.5 s per frame (approx. 0.67 FPS). This is substantially slower than the real-time requirement of 24–30 FPS, where each frame must be processed within 33–41 ms. While the current model does not yet support real-time processing, it is anticipated that accelerating the framework through parallel computation or deploying it on hardware platforms such as SoCs or FPGAs could enable real-time operation, with the goal of enabling deployment in real-time systems such as autonomous driving and intelligent surveillance.

## 5. Conclusions

In this paper, we proposed a method that integrates CycleGAN with the traditional Retinex algorithm to address sandstorm-obscured images. The approach effectively mitigates common issues such as color distortion and local information loss in dusty environments. Retinex-based preprocessing was applied to adjust brightness and enhance contrast, thereby improving the learning capability of CycleGAN. To further preserve color fidelity, the image was processed in the L channel of the LAB color space, while the A and B channels were combined with a small-scale SSR model before conversion back to the RGB space. The experimental results showed that paired training significantly outperformed unpaired training in suppressing noise and dust interference. In addition, incorporating SSR-processed images into the training dataset strengthened the model’s ability to recover object details and eliminate yellow-sand artifacts. As a result, CycleGAN trained with SSR-processed paired images was adopted as the core of the proposed framework.

Comprehensive experimental results with conventional methods demonstrated that the proposed method not only improved the clarity of sand- and dust-degraded images and emphasized local details compared to conventional algorithms, but also enhanced brightness and enriched the representation of dark regions. In particular, while conventional methods failed to completely remove the yellowish tint caused by sand, the proposed method effectively eliminated both the yellow haze and dust-related interference, thereby greatly improving the ability to represent fine details of objects. Furthermore, when compared with recent sand–dust removal methods published in the past three years—CVCGCBD (2022), ROP+ (2022), Fusion Strategy (2022), and TOENet (2023)—the proposed method achieved superior performance in terms of naturalness (BRISQUE), contrast (MCMA), and sharpness metrics (S3 and LPC-SI).

Overall, the proposed method effectively enhances local contrast, removes dust interference, sharpens details, and improves brightness in sandstorm-degraded images. From an application standpoint, it holds practical value by ensuring the stable monitoring of system operation, even under sandstorm conditions. In particular, its integration into closed-circuit television (CCTV) systems can reduce sand-induced color distortion and blurring, thereby improving visual clarity for real-world surveillance tasks and enhancing object recognition performance in adverse weather conditions where conventional sensors typically struggle.

For future work, developing novel image transformation networks beyond GAN-based approaches will be essential to address the issue of faint color representation observed during training with SSR-processed images. In addition, implementing the framework on hardware platforms will be critical to enable real-time processing for practical deployment. To overcome the inherent difficulty of constructing paired training datasets, exploring alternative architectures that can operate effectively without strict pairing requirements represents an important research direction.

## Figures and Tables

**Table 1:** Ablation study results from CycleGAN to the proposed 3-Scales SSR CycleGAN (up arrow: higher is better; down arrow: lower is better).

	Image Quality Evaluation			Image-Sharpening Evaluation	
	BRISQUE ↓	PIQE ↓	MCMA ↑	S3 ↑	LPC-SI ↑
CycleGAN (Unpaired)	19.233	26.791	0.662	0.191	0.949
CycleGAN (Paired)	24.485	29.930	0.629	0.157	0.953
CycleGAN SSR1	25.353	36.539	0.665	0.144	0.961
CycleGAN SSR10	26.727	31.758	0.661	0.213	0.966
CycleGAN SSR40	27.000	33.458	0.661	0.324	0.973
CycleGAN SSR1 + SSR10	24.562	33.182	0.689	0.210	0.968
Proposed method (SSR1 + SSR10 + SSR40)	17.238	30.360	0.700	0.288	0.973

**Table 2:** Non-reference evaluation metrics of images containing dust interference (up arrow: higher is better; down arrow: lower is better).

	Image Quality Evaluation			Image-Sharpening Evaluation	
	BRISQUE ↓	PIQE ↓	MCMA ↑	S3 ↑	LPC-SI ↑
SDIE [2]	28.636	36.660	0.671	0.148	0.955
CVCAGCBD [3]	28.351	38.454	0.661	0.167	0.9592
ROP+ [5]	27.107	40.597	0.683	0.245	0.9585
Fusion Strategy [1]	23.181	34.558	0.636	0.199	0.956
TOENet [6]	26.846	34.823	0.650	0.151	0.953
CycleGAN	19.233	**26.791**	0.662	0.191	0.949
Proposed Method	**17.238**	30.360	**0.700**	**0.288**	**0.973**

**Table 3:** Processing time and relative runtime for complexity evaluation.

	Processing Time	Runtime Factor (Method/Proposed)
SDIE [2]	0.336 s.	0.223
CVCAGCBD [3]	0.372 s.	0.247
ROP+ [5]	0.685 s.	0.455
Fusion Strategy [1]	0.512 s.	0.340
TOENet [6]	0.454 s.	0.302
CycleGAN	1.294 s.	0.860
Proposed Method	1.504 s.	1.00

## Data Availability

The original sand–dust data presented in this study are openly available in Scientific Reports at doi:10.1038/s41598-022-17530-3, reference [1].
